# Complication Profiles following Right-Sided Segmental Colectomy: A Comparative Retrospective Analysis of Crohn’s Disease and Colon Cancer

**DOI:** 10.1159/mpp/adpag010

**Published:** 2026-09-01

**Authors:** Jasim Alabbad, Noor Alfadhli, Nourah Almuhana, Fawaz Alnaqi

**Affiliations:** a Department of Surgery, College of Medicine, Kuwait University, Kuwait City, Kuwait; b Department of Surgery, Mubarak Al-Kabeer Hospital, Kuwait City, Kuwait

**Keywords:** Crohn’s disease, Colon cancer, Right-sided segmental colectomy, Complication profile

## Abstract

**Objectives:**

Concerns regarding the higher incidence of postoperative complications in Crohn’s disease patients may delay surgical resection. Calibrating postoperative risks specific to Crohn’s disease, with the more common indication of colon cancer, is valuable for making informed surgical decisions. The primary objective of this study was to report a disease-specific postoperative complication profile. The secondary objective was to identify factors associated with significant complications (Clavien-Dindo classification grade ≥2).

**Subjects and Methods:**

This retrospective study was conducted at a tertiary center from January 2010 to December 2025 on adult patients who underwent right-sided segmental colectomy for Crohn’s disease or colon cancer. The data analyzed included demographics, comorbidities, laboratory findings, and perioperative clinical variables and 30-day postoperative complications.

**Results:**

A total of 230 patients met the inclusion criteria (86 with Crohn’s disease and 144 with colon cancer). Patients with Crohn’s disease were significantly younger and had fewer associated comorbidities. No differences were observed in the overall postoperative complications. However, among patients who developed complications, the rate of infectious complications was higher in patients with Crohn’s disease compared to patients with colon cancer (72.4% vs. 46.4%, *p* = 0.022). Respiratory complications occurred only in patients with colon cancer. Chronic kidney disease and conversion to open surgery were found to be independently associated with significant complications on multivariate regression analysis.

**Conclusion:**

Overall postoperative complications rates were comparable between Crohn’s disease and colon cancer patients undergoing right-sided segmental colectomy. However, a disease-specific complication profile was noted, with a higher rate of infectious complications in Crohn’s disease.

**Highlights of the Study**

- Right-sided segmental colectomy showed comparable overall postoperative complications in Crohn’s disease and colon cancer patients.
- Crohn’s disease was associated with a higher incidence of postoperative infectious complications.
- Higher infectious complication rates did not translate into meaningful differences in short-term recovery or outcomes.

## Introduction

The understanding of the therapeutic landscape of Crohn’s disease (CD) continues to evolve as longitudinal outcome data accumulate over time. Historically managed with surgery, the focus has shifted toward medical therapy as the benefits of corticosteroids and biological agents have become more evident. Currently, CD treatment follows a multidisciplinary, stepwise approach, and surgery is typically reserved for patients in whom maximal medical therapy has been unsuccessful or when disease-related complications develop [1].

Recent literature shows that primary surgical resection for CD confined to the ileocecal region offers favorable long-term outcomes, better disease control, and cost-effectiveness compared with biological therapy [2–5]. However, early surgery is sometimes deferred because of concerns regarding increased postoperative morbidity [6]. Existing studies have primarily focused on reporting the surgical outcomes of CD independently, rather than describing outcomes in the context of other indications, such as colon cancer (CC), which is one of the most common indications for right-sided segmental colectomy (RSC). This leaves a methodological gap that limits the ability to accurately identify the complication profile unique to the inflammatory condition and leaves uncertainty regarding whether CD patients experience a distinct pattern of short-term postoperative morbidity. This study aimed to evaluate short-term postoperative outcomes following RSC in patients with CD and CC within a single-center cohort, benefiting from consistent surgical practice and detailed perioperative data. The primary objective was to determine the incidence and pattern of short-term postoperative complications, while the secondary objective was to identify factors associated with significant postoperative morbidity.

## Methods

### Study Design and Setting

This retrospective study was conducted between January 2010 and December 2025 at an academic institution in Kuwait. The Ministry of Health’s Standing Committee for Coordination of Health and Medical Research approved this study, and the requirement for written consent was waived due to the retrospective design of this study. This study adhered to the ethical standards outlined in the 1964 Declaration of Helsinki and was reported in accordance with the Strengthening the Reporting of Observational Studies in Epidemiology (STROBE) criteria [7]. The STROBE checklist is included in the online supplemental Digital Content (for all online suppl. material, see https://doi.org/10.1159/mpp/adpag010).

#### Study Participants

All consecutive patients aged ≥18 years who underwent RSC for a diagnosis of CD or CC were identified from electronic hospital records and included in the study. CD and CC diagnoses were established preoperatively using laboratory tests, imaging, endoscopy, and pathological analysis. The diagnosis was further confirmed by postoperative pathological examination of the resected specimens.

#### Exclusion Criteria

We excluded patients under 18 years of age, those who underwent RSC for an indication other than CD or CC, those with metastatic CC, those who underwent abdominal surgery without RSC, and those who underwent surgery for CC with a background history of CD.

#### Surgical Procedure

RSC was defined as ileocolic resection or right hemicolectomy. Right hemicolectomy was defined as the ligation of the ileocolic and right colic vascular pedicles with segmental bowel resection, with or without anastomosis. Ileocecal resection was defined as resection of the cecum and terminal ileum, with or without anastomosis, to a macroscopically healthy bowel.

#### Perioperative Care

Perioperative care included patient education, perioperative antibiotic administration, and venous thromboembolism prophylaxis. Postoperatively, patients were encouraged to mobilize early, initiate oral intake shortly after surgery, and receive multimodal pain relief. Patients were discharged from the hospital when they tolerated an oral diet and their pain was controlled. All patients had a scheduled outpatient follow-up visit within 1 month after discharge, and subsequent visits were scheduled as required.

### Data Collection

Data were extracted from inpatient medical records, operative registries, outpatient records, and emergency department visits. The data collected included patient demographics and preoperative characteristics, including comorbidities, baseline laboratory values, and CT scan findings. The Montreal classification system was used to describe CD phenotypes [8]. The postoperative outcome data were recorded for at least 30 days following surgery.

Clinical variables were defined by using standard criteria. Systemic steroid exposure was defined as use within 8 weeks before surgery and biological therapy exposure as use within the preceding 12 weeks. Anemia was defined as a hemoglobin level <10^9^ g/L, according to the World Health Organization (WHO) definition for moderate and severe anemia and malnutrition as a serum albumin level of <35 g/L [9]. Chronic kidney disease (CKD) was classified as Kidney Disease: Improving Global Outcomes (KDIGO) grade ≥3b (estimated glomerular filtration rate <45 mL/min) [10]. Emergency surgery was defined as a procedure performed during an unplanned hospital admission.

### Outcomes Measured

The primary outcome was to characterize and document the postoperative complication profiles following RSC in patients with CD or CC. The postoperative complications included surgical site infection (SSI), anastomotic leak, reoperation, readmission rate, and mortality, with length of stay (LOS) also reported. Anastomotic leak was defined as an anastomotic site defect with signs of peritonitis and uncontained leakage of intestinal contents that required surgery; patients who had a diverting loop ileostomy at initial surgery were excluded for leak rate calculation. Complications were stratified according to the Clavien-Dindo classification [11]. Infectious complications were a composite of complications, including SSIs, anastomotic leak, pneumonia, urinary tract infections, and bloodstream infection.

The secondary outcome was the association between perioperative factors and the development of significant complications. Significant complications were defined as Clavien-Dindo classification grade ≥2. Accordingly, the study participants were categorized based on the presence or absence of significant complications, irrespective of their diagnosis. The demographic, clinical, and operative variables were compared between the two groups.

### Statistical Methods

Continuous variables did not follow a normal distribution as determined by the Shapiro-Wilk test. Accordingly, they were presented as medians with interquartile range and compared using the Mann-Whitney U test. Categorical variables were expressed as frequencies (percentages) and compared using the chi-square or Fisher’s exact test, as appropriate. Variables demonstrating clinical relevance or statistical significance in the univariate analysis were included in a multivariate regression model to assess their independent associations with the development of significant complications. The model included seven categorical variables. Collinearity was assessed using variance inflation factors, with no variable exceeding conventional thresholds, and missing data (<5%) were handled using complete-case analysis. Statistical analyses were performed using IBM SPSS Statistics version 29.0.2.0 (IBM Corp., Armonk, NY, USA). Statistical significance was set at *p* < 0.05.

## Results

During the study period, 230 patients (86 with CD and 144 with CC) met the inclusion criteria and were included in the analysis.

### Preoperative Characteristics

The preoperative characteristics and operative details of the patients have been presented in Table 1. The median age of patients with CD was significantly lower than that of patients with CC (26.0 vs. 61.0 years, *p* < 0.001). The CC patients exhibited a higher prevalence of comorbidities, including hypertension, diabetes mellitus, stroke, and CKD. Preoperative anemia was more prevalent in patients with CC than in those with CD (60.4% vs. 22.1%, *p* < 0.001). Preoperative malnutrition was present in 41.3% of the cohort, with no significant intergroup differences. Among patients with CD, 18 (20.9%) had non-stricturing, non-penetrating disease, 31 (36.1%) had stricturing disease, and 37 (43.0%) had penetrating disease. In addition, 16 patients (18.6%) had concomitant perianal disease and 9 (10.5%) reported extraintestinal manifestations. Preoperatively, 12 (14.0%) patients were treated with steroids, 26 (30.2%) received biologics, and 9 (10.5%) patients received both steroids and biologics.

**Table 1.** Perioperative characteristics of the patients

​	CD, *n* = 86	CC, *n* = 144	*p* value
*Preoperative characteristics*			
Age, median (IQR), years	26.0 (20.0–33.3)	61.0 (49.0–70.8)	<0.001
Sex, *n* (%)	​	​	0.330
Female	32 (37.2)	63 (43.8)	​
Male	54 (62.8)	81 (56.3)	​
Associated co-morbidities, *n* (%)			
Active smokers	19 (22.1)	18 (12.5)	0.055
Hypertension	1 (1.2)	61 (42.4)	<0.001
Ischemic heart disease	0 (0.0)	4 (2.8)	0.30
Congestive heart failure	0 (0.0)	4 (2.8)	0.30
Peripheral vascular disease	0 (0.0)	4 (2.8)	0.30
Diabetes mellitus	0 (0.0)	60 (41.7)	<0.001
Stroke	0 (0.0)	7 (4.9)	0.048
CKD	0 (0.0)	8 (5 0.6)	0.027
Chronic obstructive pulmonary disease	0 (0.0)	2 (1.4)	0.530
Preoperative anemia	19 (22.1)	87 (60.4)	<0.001
Preoperative malnutrition	37 (44.6)	58 (42.6)	0.780
Preoperative steroids only	12 (14.0)	2 (1.4)	<0.001
Preoperative biologics only	26 (30.2)	0 (0.0)	<0.001
Preoperative steroids and biologics	9 (10.5)	0 (0.0)	<0.001
*Operative details*, *n* (%)			
Surgical approach	​	​	0.204
Open	10 (11.6)	27 (18.8)	​
Laparoscopic	62 (72.1)	102 (70.8)	​
Converted	14 (16.3)	15 (10.4)	​
Emergency surgery	9 (10.5)	10 (6.9)	0.348
Intraoperative drain placement	25 (29.1)	22 (15.3)	0.012
Diverting ileostomy	20 (23.3)	1 (0.7)	<0.001

IQR, interquartile range.

### Perioperative Outcomes

There was no difference between the surgical approaches; however, there was a significant utilization of diverting loop ileostomy and intraoperative drain placement in patients with CD. The postoperative outcomes of patients following RSC have been presented in Table 2. The overall complication rate was 37.0%. There was no difference in the complication rate between patients with CD and those with CC (33.7% vs. 38.9%, *p* = 0.432). In addition, there was no significant difference in the distribution of complications when stratified by the Clavien-Dindo classification. A detailed summary of the postoperative complication profiles is given in Table 3. Organ/space SSI was more frequently observed in patients with CD. In contrast, respiratory complications were observed only in patients with CC, with pneumonia being the most common event. The LOS and unplanned readmission rates were comparable. Mortality occurred in 1 patient with CC. Among patients who developed postoperative complications, the complication profile differed between diagnoses. Infectious complications were significantly more common among patients with CD than those with CC (21 patients [72.4%] vs. 22 patients [46.4%], *p* = 0.022).

**Table 2.** Postoperative outcomes

​	Total, *n* = 230	CD, *n* = 86	CC, *n* = 144	*p* value
Any complication^a^, *n* (%)	85 (37.0)	29 (33.7)	56 (38.9)	0.432
Complications by Clavien-Dindo class, *n* (%)	​	​	​	0.739
Class 1	12 (14.1)	4 (13.8)	8 (14.3)	​
Class 2	54 (63.5)	17 (58.6)	37 (66.1)	​
Class 3	14 (16.5)	7 (24.1)	7 (12.5)	​
Class 4	4 (4.7)	1 (3.4)	3 (5.4)	​
Class 5	1 (1.2)	0 (0.0)	1 (1.8)	​
Reoperation	6 (2.6)	3 (3.5)	3 (2.1)	0.674
Unplanned readmission	22 (9.6)	11 (12.8)	11 (7.7)	0.211
LOS, median (IQR), days	6.0 (4.0, 8.0)	6.0 (5.0, 8.0)	6.0 (4.0, 8.8)	0.356

IQR, interquartile range.

^a^ Defined as a patient with ≥1 complication.

**Table 3.** Complication profiles following RSC

​	Total, *n* = 230	CD, *n* = 86	CC, *n* = 144	*p* value
Anastomotic leak^a^, *n* (%)	3 (1.4)	2 (2.9)	1 (0.7)	0.252
Ileus^b^, *n* (%)	14 (6.1)	5 (5.8)	9 (6.3)	0.894
Small bowel obstruction, *n* (%)	2 (0.9)	0 (0.0)	2 (1.4)	0.530
Superficial/deep SSI, *n* (%)	24 (10.4)	10 (11.6)	14 (9.7)	0.647
Organ/space SSI, *n* (%)	15 (6.5)	10 (11.6)	5 (3.5)	0.015
Percutaneous drain insertion, *n* (%)	10 (4.3)	6 (7.0)	4 (2.8)	0.181
Blood stream infection, *n* (%)	3 (1.3)	0 (0.0)	3 (2.1)	0.295
Urinary tract infection, *n* (%)	2 (0.9)	2 (2.3)	0 (0.0)	0.139
Anastomotic site bleeding, *n* (%)	2 (0.9)	1 (1.2)	1 (0.7)	1.00
Intra-peritoneal bleeding, *n* (%)	8 (3.8)	3 (3.9)	5 (3.7)	1.00
Postoperative transfusion, *n* (%)	35 (15.2)	10 (11.6)	25 (17.4)	0.242
Stroke, *n* (%)	2 (0.9)	0 (0.0)	2 (1.4)	0.530
Cardiac arrhythmias^c^, *n* (%)	4 (1.7)	0 (0.0)	4 (2.8)	0.300
Venous thromboembolism, *n* (%)	3 (1.3)	1 (1.2)	2 (1.4)	1.00
Respiratory complications^d^, *n* (%)	11 (4.8)	0 (0.0)	11 (7.6)	0.009
Acute kidney injury, *n* (%)	5 (2.2)	0 (0.0)	5 (3.5)	0.160

^a^ Excluding patients with diverting ileostomy.

^b^ Defined as the inability to pass gas for > 5 days or the need for nasogastric tube insertion.

^c^ Includes supraventricular tachycardia, atrial fibrillation, and symptomatic bradycardia.

^d^ Includes pneumonia, atelectasis, respiratory failure, and pleural effusion.

### Factors Associated with Complications

Upon stratifying the cohort into patients with and without complications, it was found that female sex, diabetes mellitus, CKD, and malnutrition were significantly associated with higher complication rates. Moreover, patients who underwent emergency surgery and those who required conversion from laparoscopic to open surgery experienced higher complication rates. As expected, patients with significant complications had a longer median LOS (7.0 [5.0–11.5] vs. 6.0 [4.0–7.0] days, *p* < 0.001). The factors associated with significant complications in the study population have been presented in Table 4. In the multivariate regression analysis, CKD and conversion to open surgery were independently associated with postoperative complications (Table 5).

**Table 4.** Factors associated with the development of significant complications (Clavien-Dindo classification grade ≥ 2)

​	No complication, *n* = 157	Complication, *n* = 73	*p* value
Diagnosis	​	​	0.501
CD	61 (38.9)	25 (34.2)	​
CC	96 (61.1)	48 (65.8)	​
Age, median (IQR), years	45.0 (27.0–63.0)	50.0 (33.0–67.0)	0.134
Sex, *n* (%)	​	​	0.049
Female	58 (36.9)	37 (50.7)	​
Male	99 (63.1)	36 (49.3)	​
Associated co-morbidities, *n* (%)			
Active smokers	25 (15.9)	12 (16.4)	0.921
Hypertension	37 (23.6)	25 (34.7)	0.078
Ischemic heart disease	2 (1.3)	2 (2.7)	0.593
Congestive heart failure	2 (1.3)	2 (2.7)	0.593
Peripheral vascular disease	1 (0.6)	3 (4.1)	0.096
Diabetes mellitus	34 (21.7)	26 (35.6)	0.025
Stroke	4 (2.5)	3 (4.1)	0.682
CKD	2 (1.3)	6 (8.2)	0.014
Chronic obstructive pulmonary disease	2 (1.3)	0 (0.0)	1.00
Preoperative anemia	69 (43.9)	37 (50.7)	0.340
Preoperative malnutrition	57 (38.3)	38 (54.3)	0.026
Preoperative steroids only	8 (5.1)	6 (8.2)	0.382
Preoperative biologics only	16 (10.2)	10 (13.7)	0.503
Preoperative steroids and biologics	6 (3.8)	3 (4.1)	1.00
Operative details			
Emergency surgery	9 (5.7)	10 (13.7)	0.041
Surgical approach	​	​	0.020
Open	23 (14.6)	14 (19.4)	​
Laparoscopic	120 (76.4)	44 (60.3)	​
Converted	14 (8.9)	15 (20.5)	​
Intraoperative drain placement	32 (20.4)	15 (20.5)	0.977
Diverting loop ileostomy	11 (7.0)	10 (13.7)	0.101

IQR, interquartile range.

**Table 5.** Multivariate regression analysis of factors associated with significant complications (Clavien-Dindo classification grade ≥2)

​	OR	95% confidence interval		*p* value
		lower	upper	
Crohn’s diagnosis	1.144	0.544	2.404	0.722
Female sex	1.603	0.652	2.959	0.132
Diabetes mellitus	1.904	0.883	4.105	0.100
CKD	5.996	1.088	33.060	0.040
Preoperative malnutrition	1.565	0.843	2.908	0.156
Emergency surgery	1.941	0.647	5.819	0.237
Surgical approach*				
Open surgery	1.278	0.537	3.044	0.580
Laparoscopic to open surgery	2.679	1.130	6.348	0.025

Reference: laparoscopic surgery. The surgical approach was the only non-binary variable and was modeled as a three-level categorical variable.

## Discussion

In this study, we report the outcomes following RSC in patients with CD and those with CC, each characterized by unique baseline features. Although the overall complication rates were similar across the cohort, these baseline characteristics appeared to drive the contrasting postoperative complication profiles. Patients with CD were younger and had minimal baseline comorbidities. However, their ongoing exposure to immunosuppressive therapy until the time of surgery underscores the chronicity of CD and the degree of underlying immune compromise, which is reflected in the complications experienced by patients. In contrast, patients with CC were nearly 3 decades older and exhibited a greater burden of chronic medical conditions, including diabetes mellitus, hypertension, and preoperative anemia. These findings highlight the influence of disease-specific factors on the preoperative risk profile, aligning with reported trends [12, 13].

Patients with CD exhibited a different complication profile. Notably, organ/space SSI occurred more frequently in patients with CD, a pattern that likely reflects the cumulative impact of the disease and associated treatment. Pneumonia occurred exclusively in patients with CC, possibly reflecting their older age and greater comorbidity burden. Our findings align with those of Larson et al. [12] who analyzed NSQIP data from 17,500 patients and similarly reported higher rates of surgical complications in CD and more medical complications in CC, including respiratory complications. Conversely, a single-center retrospective study by Grass et al. [14] did not find statistically significant differences in postoperative outcomes between patients with CD and those with CC, although their data showed a numerical trend toward more systemic complications in patients with CC. An in-depth analysis of the CD cohort revealed unique features of the surgical indication that further explains the observed higher rate of infectious complications, particularly organ/space SSI. Nearly 40% of patients required surgery for penetrating disease, which inherently carries a risk of localized contamination, further compounding the effect of CD on postoperative infectious risk. Despite the higher infection rate, the need for postoperative percutaneous drainage did not differ between patients with CD and those with CC. Most cases were successfully managed with antibiotic therapy alone, indicating that while patients with CD may experience more organ/space SSI, this did not translate into a greater requirement for invasive postoperative interventions. Additionally, these differences did not translate to other postoperative outcome measures, including higher rates of reoperation, readmission, and LOS.

In this study, the overall anastomotic leak rate was 1.4%, and patients with CD did not show a statistically significant higher leak rate. The published anastomotic leak rates following RSC show considerable variability, with some series reporting rates as high as 9% [15, 16]. Moreover, evidence from previous observational studies has been inconsistent when considering disease-specific leak rates. A retrospective study by Tiberi et al. [17] and the European Society of Coloproctology (ESCP) collaborating group [13] reported comparable leak rates in CD and CC, while others [12, 18] identified CD as being associated with higher leak rates. This inconsistency may be attributed to differences in study design, patient selection, and the definition of a leak across studies [19]. While no difference was present in leak rates, it is important to note that in this study, 23.3% of patients with CD were diverted and excluded from the leak rate analysis. It is challenging to retrospectively examine the reasons for diversion in these patients; however, factors such as disease chronicity, malnutrition, and the use of steroids or biological therapy generally influence this decision. Thus, anastomotic outcomes should be interpreted cautiously.

Two recent studies of postoperative outcomes after RSC in CD and CC reported broadly similar outcomes but differed in methodology and the disease-specific postoperative pattern observed in the present study. Choi et al. [20] conducted a large retrospective cohort study excluding patients with diverting stoma and found no significant differences in postoperative outcomes. A retrospective study by Bursztyn et al. [21] reported a higher anastomotic leak rate in CD. However, unlike the present study, their stoma rates were lower, which may account for the higher leak rates in patients with CD.

In addition to the disease-specific complication profile, it is important to consider the perioperative factors associated with the risk of developing significant complications. In our cohort, female sex, diabetes mellitus, CKD, preoperative malnutrition, emergency surgery, and conversion to an open procedure were associated with the development of significant complications in both CD and CC groups. These associations are consistent with previous reports identifying malnutrition, diabetes, and CKD as predictors of adverse outcomes [22–24]. Interestingly, we did not find an association between preoperative anemia and postoperative complications, a factor implicated in other studies [25]. Furthermore, in the multivariate regression analysis, only CKD and conversion to open surgery demonstrated a significant association with complications, although the wide confidence interval warrants cautious interpretation.

Historically, surgery for CD has been deferred until late in the disease course, largely because of concerns about postoperative morbidity and the perception that operative intervention represents a failure of medical therapy [6]. However, evidence supports early bowel resection as a primary treatment for localized ileocolic CD, demonstrating outcomes comparable to those achieved with the initiation of biological treatment [4, 5]. The findings of this study provides a detailed postoperative complication profile of patients with CD who underwent RSC. Understanding how these outcomes compare with more common RSC indications enables surgeons to anticipate postoperative risks more accurately, strengthen the quality of preoperative counselling, and reduce unwarranted deferral of surgery.

The limitations of this study are primarily attributed to its single-center retrospective design, which may introduce selection bias, constrain its ability to establish causal relationships, and limit its generalizability of the results. The inherent complexity of CD, characterized by its heterogeneous clinical presentations, results in variable thresholds for surgical intervention, which are largely dictated by the underlying indications. This heterogeneity introduces significant challenges in interpreting postoperative outcomes and underscores the need for individualized surgical decision-making in this patient population. Despite these limitations, this study offers valuable insights through a detailed retrospective analysis. Future research should address these issues through large-scale, prospective studies.

## Conclusion

The findings of this retrospective study suggest that RSC is associated with broadly comparable overall postoperative outcomes in patients with CD, although CD was associated with higher overall infectious complications. This did not appear to result in meaningful differences in overall recovery and short-term postoperative outcomes. Leak rate warrants cautious interpretation as a significant proportion of CD patients were diverted.

## Statement of Ethics

The institutional Ethical Committee of the Ministry of Health approved this study, and the requirement for written consent was waived due to the retrospective design of this study.

## Conflict of Interest Statement

All authors certify that they have no affiliations with or involvement in any organization or entity with any financial or non-financial interest in the subject matter or materials discussed in this manuscript.

## Funding Sources

The authors did not receive funding or support from any organization for the submitted work.

## Author Contributions

Study conception and design, acquisition of data, drafting of manuscript, and critical revision of manuscript: Jasim Alabbad, Noor Alfadhli, Nourah Almuhana, and Fawaz Alnaqi. Analysis and interpretation of data: Jasim Alabbad and Fawaz Alnaqi.

## Data Availability

The data supporting the findings of this study are not publicly available due to privacy and institutional restrictions. Access to the dataset can be provided upon reasonable request to the corresponding author, subject to approval by the local Institutional Review Board committee.
